# Rethinking the Design of Low-Cost Point-of-Care Diagnostic Devices

**DOI:** 10.3390/mi8110317

**Published:** 2017-10-27

**Authors:** Faith W. Kimani, Samuel M. Mwangi, Benjamin J. Kwasa, Abdi M. Kusow, Benjamin K. Ngugi, Jiahao Chen, Xinyu Liu, Rebecca Cademartiri, Martin M. Thuo

**Affiliations:** 1 Kiambu District Hospital, Kiambu 00900, Kenya; kimani.faith@gmail.com; 2 School of Public Health, Kenyatta University, Nairobi 00100, Kenya; 3 Department of Sociology, Kenyatta University, Nairobi 00100, Kenya; 4 Department of Aerospace Engineering, Iowa State University, Ames, IA 50011, USA; bjkwasa@iastate.edu; 5 Department of Sociology, Iowa State University, Ames, IA 00100, USA; kusow@iastate.edu; 6 Department of Information Systems and Operations Management, Suffolk University, Boston, MA 02108, USA; bngugi@suffolk.edu; 7 Department of Material Science and Engineering, Iowa State University, Ames, IA 50011, USA; jiahao@iastate.edu; 8 Department of Mechanical Engineering and Industrial Engineering, University of Toronto, 5 King’s College Road, Toronto, ON M5S 3G8, Canada; xyliu@mie.utoronto.ca; 9 Department of Chemical and Biological Engineering, Iowa State University, Ames, IA 50011, USA; 10 Center for Bioplastics and Biocomposites (CB2), Iowa State University, Ames, IA 50011, USA

**Keywords:** low cost, diagnostics, technology adoption, value-added design, health care, rapid diagnostics

## Abstract

Reducing the global diseases burden requires effective diagnosis and treatment. In the developing world, accurate diagnosis can be the most expensive and time-consuming aspect of health care. Healthcare cost can, however, be reduced by use of affordable rapid diagnostic tests (RDTs). In the developed world, low-cost RDTs are being developed in many research laboratories; however, they are not being equally adopted in the developing countries. This disconnect points to a gap in the design philosophy, where parameterization of design variables ignores the most critical component of the system, the point-of-use stakeholders (e.g., doctors, nurses and patients). Herein, we demonstrated that a general focus on reducing cost (i.e., “low-cost”), rather than efficiency and reliability is misguided by the assumption that poverty reduces the value individuals place on their well-being. A case study of clinicians in Kenya showed that “zero-cost” is a low-weight parameter for point-of-use stakeholders, while reliability and standardization are crucial. We therefore argue that a user-driven, value-addition systems-engineering approach is needed for the design of RDTs to enhance adoption and translation into the field.

## 1. Background

Affordable and accessible health care is a major challenge to national development. For example, in Kenya, 51% of health care is paid for out of pocket, and 46% of the population lives on US $1.00 or less per day [1,2]. This combination of low income and high out-of-pocket costs for healthcare makes it imperative to keep overall health-care costs low. Health care consists of three interlinked areas: disease prevention, diagnosis and treatment. Disease prevention and treatment have received much attention recently with widespread immunization and treatment programs organized by international organizations such as the World Health Organization (WHO), but advances in affordable disease diagnostics have lagged behind leading to an over-reliance on clinical symptoms [3]. The reliance on symptoms is partly attributed to the fact that many laboratories in developing countries are not well equipped [4] and lack well-maintained equipment and/or skilled laboratory personnel.

The dire need for better health care among the world’s less-fortunate has garnered the attention of scientists and engineers in the developed world [5], who are developing rapid diagnostic tests (RDTs) with a current rise in paper-based devices, with long shelf lives that do not rely on advanced equipment or trained personnel and can supplement clinical symptom diagnosis based on different materials and detection modalities [5,6,7,8,9,10,11,12,13,14]. Over the last four decades, RDTs for common diseases such as HIV [15] and malaria [16] have been developed, and some have been adopted in the field. A number of reports on recent developments in RDTs have appeared in the literature [17,18,19,20,21,22,23,24,25] but are beyond the scope of the current report. Despite the rapid increase in the number of RDTs studies, Whitesides and co-workers observed a huge discrepancy in the number of published papers on laboratory studies versus field testing and adoption [5]. Miller et al. also noted that, of the RDTs that have recently been developed in research laboratories, few have been adopted by the target end users [26].

A number of studies have shown that although RDTs can significantly reduce disease prevalence and improve the well-being of populations in the developing world [27,28,29], they have not been widely adopted. The use and impact of RDTs across the world, for example in Nigeria [30], Uganda [31], France [32], Tanzania [33], and Ghana [34], is complicated by user perceptions. All studies reported both positive (e.g., better clinical diagnosis) and negative (e.g., increased workload, reliability, and cost) perceptions of RDTs, complicating technology adoption.

**Figure 1:**
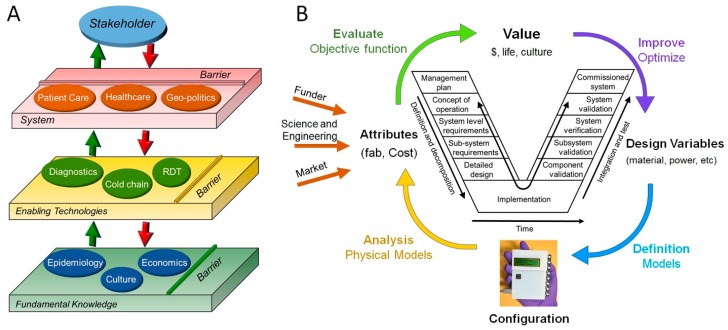
An expanded view of the challenges and approaches to effective design of affordable RDTs. (**A**) Three tier schematic diagram of the different critical levels in the design process with their associated barriers. Tier 1 captures all fundamental knowledge associated with diagnostics and healthcare, while Tier 2 is the enabling technologies that are then translated to Tier 3 here capturing the local health care system and associated socio-cultural structures. All three tiers make the overall system. (**B**) Systems engineering approach to design of low-cost RDTs with the capture capturing the underlying V-model but specifically focusing on value addition where “value” is dictated by each stakeholder but with the end-user definition carrying a higher weight. Attributes are derived from the stakeholders and the drivers of the design and fabrication (abbreviated “fab”) and cost. The existence of feedback loops during the design process allows for efficiency and appropriateness in the design, production and adoption of the product.

Technology adoption, from development to use, is a challenge, even when the technology has definite benefits for potential users [35]. The technology acceptance model (TAM) argues that behavioral intentions to use a new technology depends on its perceived usefulness and perceived ease of use [36]. Low-cost RDTs must, therefore, affirm to users their value before gaining their trust [37,38]. Trust, however, is two-tiered: (i) Confidence in the goodwill of others not to cause harm to a vulnerable person. For most developing countries, this trust is complicated by colonial history and effect of prior experiences with international development programs. (ii) Trust in the technology to better the quality of life of the user—which RDTs must gain if they are to translate from a research curiosity to use [39,40,41,42,43]. Trust in a technology can be eroded by inaccuracies and repeated failures, making it important for developers to learn what challenges users encounter and how they relate to the adoption of the technology. To ensure continued trust in a technology, iterative development is often desired where the user informs newer versions of the technology-design. This information, from all stakeholders, is combined into a technology development system, which in turns informs which enabling technologies are best suited for the particular market or challenge. It is therefore imperative that constant communication between technology developers and end-use occur if the problem is to be adequately addressed. The developed technologies, however, are based on fundamental scientific, business, socio-cultural, and geo-political knowledge. Increasing the quality of this fundamental knowledge will make more technologies available (increase competition and choice), which can lead to an increase in adoption by the end-user (key stakeholders) as long as their values are met (Figure 1A). The design challenge can, therefore, be broken down into three tiered structures: the fundamental knowledge base, integration of this information into enabling technologies, and finally the field (point-of-use healthcare and business environment), i.e., the users’ system (Figure 1A). Complexity in each of the micro-systems challenges the technology developers, and, we believe, is the basis of poor translation of many otherwise well designed enabling technologies. Besides, each tier has its own challenges, with the fundamental knowledge being complicated by the diverse nature of data (from physical and social sciences), enabling technologies being limited by state-of-the-art while diversity in culture and political landscape makes comprehension of health care systems challenging. For example, while the west relies on individual health insurance, in Kenya, for example, the community acts as the safety net through so called “*harambees*”—public fundraising events or the social responsibility of relatives to each other. It is therefore difficult to define the buying power of a healthcare market without understanding the potential (value) of life in these communities and associated micro-communities.

The challenge of understanding complicated macro-systems and design environments is, however, not new. System engineering provides a rigorous approach to the conception, design, manufacturing and retiring (end-of-life management) of technology in otherwise complex systems. This approach is made possible by use of various frameworks within systems engineering such as the V-model (insert Figure 1B), the waterfall model and the spiral model needed to generate and keep track of goals and progress throughout the lifetime of an engineering endeavor [44]. The models are particularly useful in obtaining the requirements in a system, communicating goals to designers and to measure progress. These requirements also represent the stakeholders’ desires. These models have also demonstrated the importance of end-user decision-making early in the design process especially for a complex system [45]. Value-Driven Design (VDD) provides a platform for capturing these desires and to communicate them throughout the development process (Figure 1B) [46]. At the pinnacle of this model is integration of “value” into the design process. Bloebaum and co-workers [45,46] have explained this model elsewhere, a task beyond the scope of this report. The VVD model ensures that the outputs—systems or products—are elegant solutions to existing problems that stakeholders will adopt. For RDTs to be adopted, their design cannot solely be based on the designers’ assumptions, but requires input from the stakeholders about all barriers and challenges to the effective adoption and trust in the technology. End user input is therefore critical. To ascertain the validity of this inference, we explored the perception of clinicians in Kenya with the goal of understanding some of the challenges and blind-spots in the design process.

## 2. Case-Study: Barriers to Adoption of Low-Cost RDTs in Kenya

Based on the VDD model, and a desire to design for translation/adoption, we sought to understand the most critical variables for an effective adoption of RDTs in Kenya. In Kenya, medical tests are generally ordered by clinicians (i.e., physician assistants—so-called clinical officers—and medical doctors). Because the clinicians decide which tests are done and patient generally trust a clinician’s judgment, we hypothesized that the opinion of the clinicians is representative of the point-of-care stakeholders (end-users) with regards to RDTs. This study had two goals: (1) to determine why RDTs are not adopted en mass by target users in the developing world; and (2) how to improve the design to promote adoption. We hypothesized that limited involvement of developing-world users (e.g., clinicians, academicians, policy makers, and entrepreneurs) in the design and validation of these RDTs in the developed nation’s laboratories is a potential reason for their poor translation from the western laboratories to the point of care. Although self-reporting is less accurate than direct observation, this study was designed to provide baseline results and elucidate the general factors behind the inertia in adopting RDTs. We investigated the self-reported knowledge and opinions of Kenyan clinicians about RDTs across different hospital tiers and across three different economic zones: urban, suburban, and rural–urban. This sample is representative for clinicians across most of the country. The general awareness and familiarity with RDTs among clinicians is high: There were a total of 123 valid responses from the clinicians, with 58 responses each from high- and mid-tier hospitals and only seven from the lowest-tier hospitals; the latter is because of the structure of the Kenyan health-care system. A lack of clinical officers and medical doctors in rural low-tier hospitals made it impossible to obtain a statistically significant sample from this tier. The targeted hospitals in the rural–urban zone, however, offered insight into the use of RDTs in rural settings because these hospitals are the local referral centers for rural clinics.

## 3. Results and Discussion

Upon acceptance of an invitation to participate in this study, feedback was obtained in a maximum of two days. Qualitative analysis was done through thematic coding of the open-ended responses, classifying and summarizing the information, and presenting it in descriptive form. The validity and reliability of the perceptions scale were established using the Cronbach’s alpha (0.81–0.93 for usability and reliability, and 0.23 for adoptability) [43]. All experimental and data analysis details are given in the supporting information.

We decided not to consider responses from the lowest tier for statistical analysis due to the small sample size; however, comparative qualitative data were considered.

**Figure 2:**
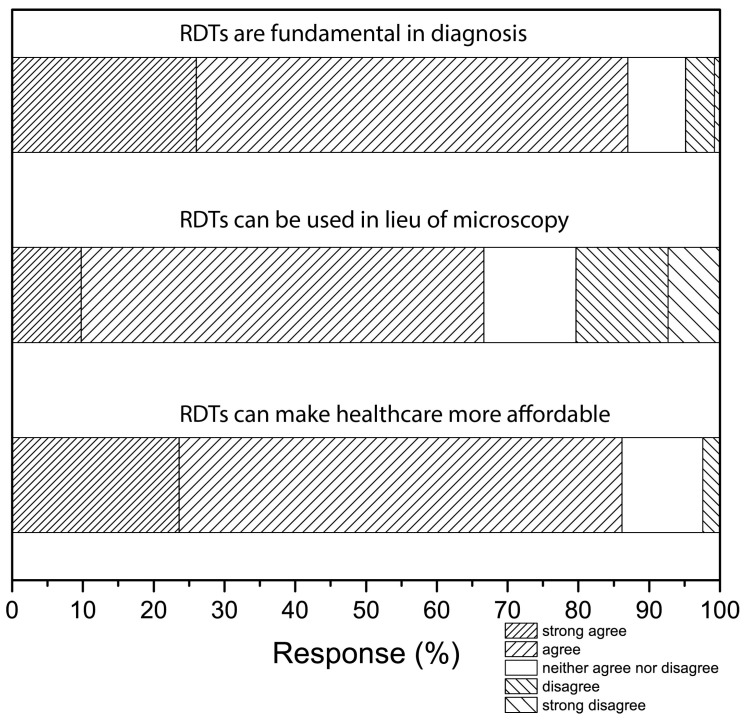
Response to qualitative questions, with the question re-phrased on top of the responses. Affirmative responses are given on the left, while negations are given on the right. The unmarked regions represent number of neutral responses.

**Figure 3:**
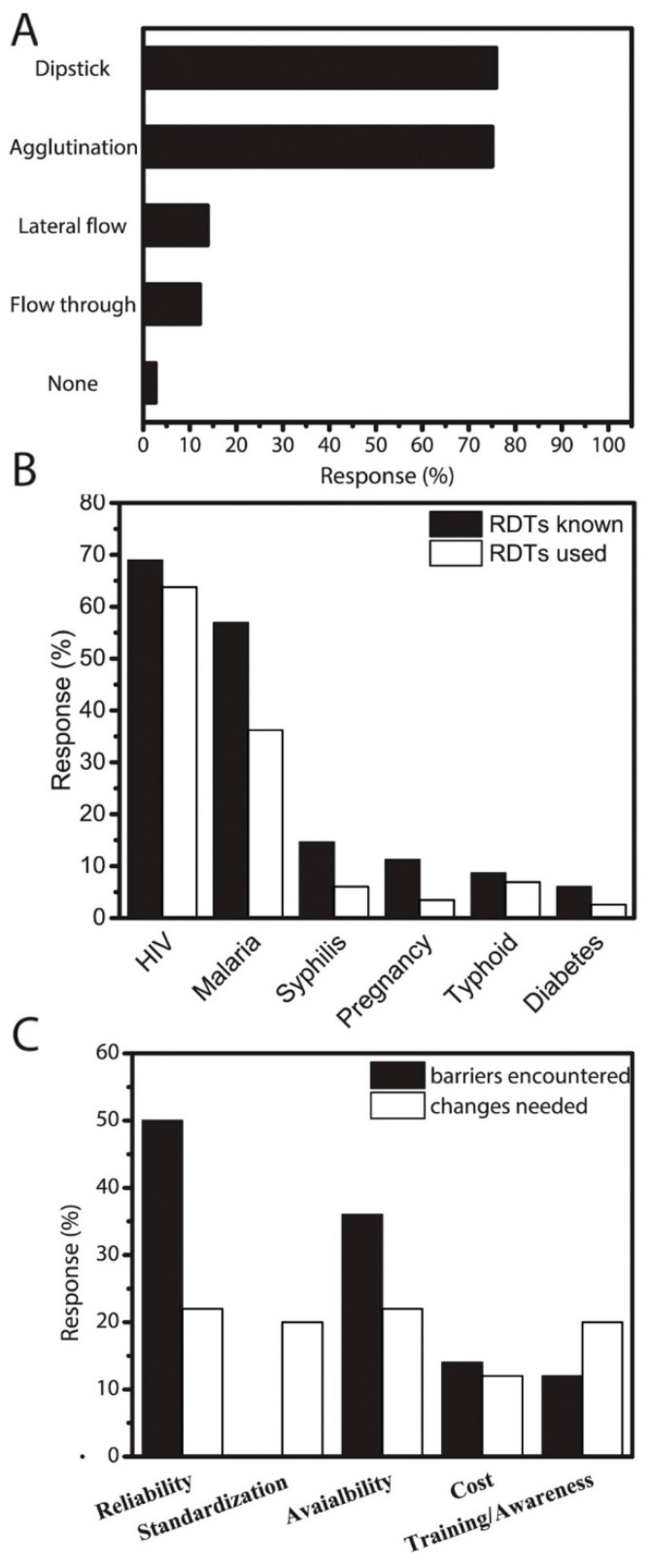
Feedback on knowledge, use and challenges in adoption of RDTs: (**A**) knowledge about specific technologies for RDTs; (**B**) percentage of clinicians that had heard about (black) or used (white) RDT for a specific disease—multiple answers possible; and (**C**) barriers encountered and perceived changes needed in RDTs. The large difference in “reliability” comes from doctors indicating both reliability and standardization, which will make results more reliable, as a change to unreliable RDTs.

All respondents indicated familiarity with RDTs, and a majority (86%) affirmed that RDTs are fundamental for disease diagnosis in Kenya with speed of the tests being their main reason (Figure 2). Clinicians affirmed that RDTs are essential in improving diagnosis and healthcare delivery in the country (Figure 2). The respondents also indicated that RDTs can replace advanced techniques such as microscopy where appropriate (Figure 2). The obvious advantages of RDTs in the Kenyan healthcare systems were also evident from the acknowledgement that these devices have the potential to make healthcare more affordable in Kenya (Figure 2). The surveyed Kenyan clinicians are conversant with various RDTs (Figure 3A), therefore knowledge of the technology was not a primary reason for the slow translation. Familiarity not only with the specific tests but also with the general overarching technologies can increase RDT adoption by decreasing natural resistance to new experiences. To determine familiarity, the clinicians were asked which specific RDT technologies they had used. A majority indicated solid phase/dipstick and agglutination tests (73% each), and a smaller proportion (<15%) identified lateral flow or flow-through devices (Figure 3A). Only a very small fraction had no knowledge of any of the above tests (2%). These data confirm that perceived usefulness, knowledge, and familiarity, are not the critical barriers in adoption of affordable RDTs. For clarity, a summary of the qualitative data (Figure 2) in the form of pie charts is also provided in the Supplementary Materials, alongside the level of training the clinicians had attained (Figures S1 and S2).

Knowledge about diseases that can be tested for with RDTs is also not a critical barrier (Figure 3B). Most clinicians (95%) named at least one disease (with 71% naming 2, and 24% naming ≥3), while a minority (5%) could not name any disease that used RDTs for diagnosis. When comparing this knowledge to whether they had used these tests themselves, it was surprising that only about 10% of the respondents that knew of ≥3 diseases that can be diagnosed with RDTs had applied them in their practice. Although a majority (81%) had diagnosed one or two diseases with an RDT, 11% had never used one. Malaria and HIV, as expected [46,47], were the most common applications for RDTs, although syphilis, pregnancy, typhoid, and diabetes were mentioned (Figure 3B). Doctors from socio-economically well-off hospitals demonstrated overall better knowledge and higher usage of RDTs in HIV testing compared to their rural counterparts. For malaria, however, there was a large discrepancy between RDT knowledge (57%) and use (36%), irrespective of the tier of the hospital. There are multiple possible reasons for this discrepancy: (1) RDTs may not be perceived as being useful for malaria diagnosis—since symptoms manifest very strongly and the spike in disease cases is predictable based on the season; (2) RDTs might not be available to clinicians; (3) RDTs may not complement other diagnostic approaches; or (4) the clinicians may have great success with other more rapid diagnostic approaches, such as identifying symptoms. For all other conditions, knowledge about the existence of RDTs for diagnosis was 1.25 to 3.25 times higher than their clinical use.

## 4. What Are the Main Barriers to Adopting RDTs?

Inasmuch as adopting a technology is related to trusting it, encountering barriers while using RDTs is detrimental to their adoption- erodes trust in the technology. Clinicians indicated that their patients were satisfied with the results derived from RDTs (97%), and would recommend RDTs for use in the future (96%). When the clinicians were asked if they thought that RDTs gave reliably accurate results, however, only half (50%) were affirmative, whereas almost half (46%) were unsure about these tests and a small proportion (4%) thought of them as inaccurate. This result is consistent with the finding that only 35% of the clinicians withheld medication when a patient’s test result was negative and that only 20% relied solely on the tests—most clinicians who gave a written reason for their answer mentioned the need to complement RDT results with the observation of clinical symptoms.

More than half of the respondents (54%) reported encountering barriers to using RDTs, and the likelihood of encountering a barrier increased as the hospital tier decreased (45% in the highest-tier hospitals and 53% in mid-tier hospitals). Of those who had encountered barriers, half (50%) indicated reliability (false positives and/or false negatives) as a major obstacle, which is one possible reason these clinicians might prescribe medication to patients with negative test results (Figure 3C). Availability of the tests or their components (46%) was the second major obstacle (Figure 3C). Only a small group considered cost (14%) or the lack of awareness/training (12%) to be major barriers. To add the point-of-use stakeholders to the system and inform the design of RDTs for increased adoption, clinicians were asked to suggest changes that are needed to increase RDT use. Nearly half (44%) suggested improving the tests themselves, with 22% suggesting improving reliability, and 20% suggesting standardization—the latter was not listed as a possible barrier in the questionnaire but they wrote it in. Increased availability was another needed change (22%), followed by awareness and training (20%); the smallest (12%) consideration was given to the tests’ costs (Figure 3C). Despite the perceived challenges and needed improvements, a majority (85%) of the clinicians sampled agreed that RDTs can make health care more affordable in Kenya (Figure 2).

**Figure 4:**
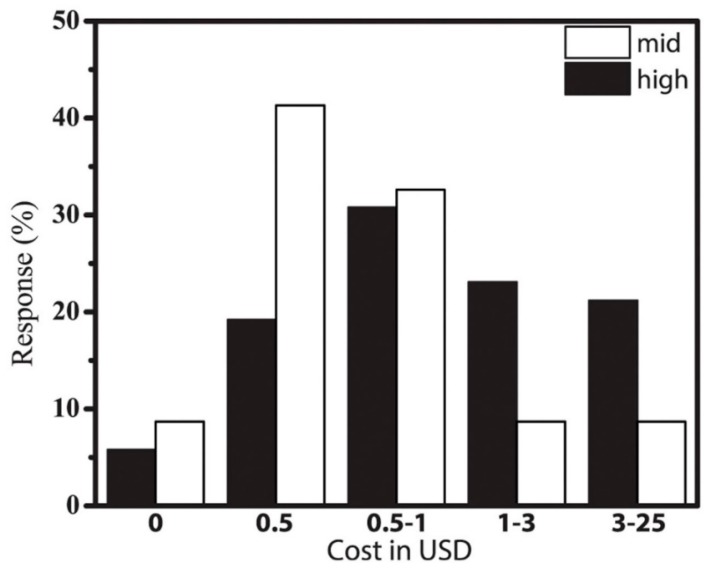
Survey of acceptable cost as given by the clinicians. Percentage of clinicians who named a specific price, depending on the level of the hospital. White: mid-tier; black: highest tier.

With the discrepancy between the focus on low-cost or even “zero-cost” in the developed world laboratories and the low perception of cost as a needed change in the developing world, there is a need to rethink the design strategies for RDTs. A race to zero-cost diagnostics seems unnecessary, especially when it is combined with a decline in the quality of RDTs. Besides, socio-cultural history (primarily colonialism and a perceived social engineering [48,49]) amongst Africans, imposes an inherent mistrust of zero-cost products, especially when such products are from the developed world. Increasing community participation in an individual’s care (through public fund-raising i.e., *harambee)*, offers an unprecedented high-level of “insurance” that allows an individual to go for quality care beyond what they can afford based on their daily earnings. It is therefore possible that a focus on an individual’s daily income as a design variable is an invalid consideration, but rather the perceived “threat” of a disease should inform the RDT designer. Surprisingly, the doctors confirmed this observation by indicating that a cost of ~$1 (mean suggested costs of $0.5 ± $0.3 (mid-tier) to $0.7 ± $0.3 (high tier)) was appropriate (Figure 4). The full range of suggested values were extremely spread out with some outlier suggestions ranging from free ($0, mostly mid-tier) to $25 (mostly high-end hospital clinicians) as captured by the data spread (Figure 4). It is therefore clear that advocacy for zero-cost diagnostic tools [6,7,8,9] will not likely increase adoption, at least in the Kenyan case, while increasing reliability and standardization can. This data also indicate that device complexity can be slightly increased to ensure reliability, standardization and ease of use.

## 5. Conclusions: What We Learned

The observed unreliability of RDTs is a major drawback to their use and can lead to low-quality services and loss of trust in these tools. This study identified self-reported mistrust of RDTs in Kenya due to consistent unreliability, and hence low perceived usefulness. To regain the clinicians’ trust, RDT development must focus on the tests’ efficiency, accuracy, and reliability, and more studies are needed to determine the importance of each of these factors during development. Establishing analytical control (standardization) while also increasing availability appeared to be more desirable to the clinicians than a focus on very low cost devices, especially when the low prices are accompanied with the loss of accuracy, reliability, and availability. The Kenyan clinicians’ responses suggest that, although the populations may not be wealthy, they highly value their health, to the extent that they are willing to spend more than a day’s wage on diagnosis. This conclusion, however, is from the doctor’s perspective and should be corroborated by the patients.

We further considered the engineering aspects of RDTs, particularly in the form of the device technology. The answers in our study indicated that simple technologies, such as dipstick and agglutination tests, were better known than more complicated ones. Although developing new RDTs with technologies that are more familiar to the intended user can lead to more rapid adoption of these methods, the low trust in the existing tests must be considered. It is therefore critical that those involved in RDT design (low-cost platforms such as paper-based devices) consider integrating a team from the target market/healthcare system if adoption and translation is critical in the design. The current norm of taking the already developed devices for “testing” in the field is bound to fail unless the end-user is integrated as part of the device development team [47,50,51].

## 6. Outlook: New Design Paradigm

Instead of largely focusing on cost, RDT designers and developers need to consider all aspects of the systems variables to produce affordable and reliable (not necessarily low-cost) diagnostic tools in which all stakeholders dictate the desired device attributes (Figure 1B). To determine what these aspects are, and how they are weighted by the doctors and patients, we propose adoption of Value-Driven Design (VDD) models in a systems engineering approach with the end-user playing an early role [45]. This approach embeds the weighted value of a technology to the patient’s life and reflects the socio-cultural background which it is being designed for. For this approach, we need input from all stakeholders including developers, policy makers, manufacturers, and, most importantly, doctors and patients at the point-of-use. We need to understand how to best “provide required resources and information for people in low- and middle-income economies in their voyage” [47] toward good health. Only an integrated systems design approach will increase the adoption of RDTs in developing countries, by eliminating the type of dissonance observed in the current study. Whereas we propose a new design paradigm, significant advances have been made in the technology development and development of fundamental knowledge in diagnosis (Figure 1A, Tier 1 and Tier 2). What is lacking, however, is an integration of this information into a diagnostic kit that is user-inspired.

## Supplementary Materials

The following are available online at www.mdpi.com/2072-666X/8/11/317/s1, Experimental and data analysis details, Figure S1: Summary data on the overall knowledge and level of training of the clinicians, Figure S2: Pie-chart summary of qualitative responses data that are also provided in Figure 2.
