# Supplementary material for: Rethinking the Design of Low-Cost Point-of-Care Diagnostic Devices
Source: Micromachines (Basel). 2017 Oct 27;8(11):317. doi: 10.3390/mi8110317 (PMC6190021; doi:10.3390/mi8110317)
Supplement: Supplementary file 1 [file micromachines-08-00317-s001.pdf]

# Rethinking the Design of Low-Cost Point-of-Care Diagnostic Devices

**Faith W. Kimani, Samuel M. Mwangi, Benjamin J. Kwasa, Abdi M. Kusow, Benjamin K. Ngugi, Jiahao Chen, Xinyu Liu, Rebecca Cademartiri and Martin M. Thuo**

## Materials and Methods

Our purpose in this study was to describe and document perceptions of Kenyan healthcare workers' acceptance and usage of RDTs. The data used in this study were collected at three study sites: Nairobi (urban), Rift Valley (rural), and Central (suburban), comprising both public and private healthcare facilities. These institutions reflect multilayered healthcare services and specializations, offering variability in perceptions and decisions to use RDTs when diagnosing diseases and illnesses. The study mainly targeted medical doctors and clinical officers in a variety of medical specializations and subspecialties from the three study sites. Our study drew a probability sample of 385 healthcare workers using a combination of sampling techniques. We used stratified sampling to draw the health facility—public and private—from a sampling frame of all hospitals in the regions, proportionate sampling was used to obtain samples from each of the three regions, and simple random sampling was used to draw the study participants from a list of all qualified doctors and clinical officers from the selected facilities. Data were collected using a semi-structured interviewer-administered questionnaire for both quantitative (closed-ended) and qualitative (open-ended) inquiries. The validity and reliability of the perceptions scale were established using the Cronbach's alpha (0.81–0.93 for usability and reliability, and 0.23 for adoptability).

Qualitative analysis was done through thematic coding of the open-ended responses, classifying and summarizing the information, and presenting it in descriptive form. The permit to conduct this study was obtained from Kenya's National Council of Science and Technology (NACOSTI). Ethical clearance was obtained from the Ethical Review Committee of Kenyatta University to collect data from human subjects. Consent was given by the participants, and they were informed that the information they provided would be used for research purposes only.

## Measures

The primary measures used to explore perceptions of Kenyan healthcare workers' acceptance and usage of RDTs were derived from a number of variables organized into three types: 1) knowledge of RDTs, 2) barriers to improving use of RDTs, and 3) the cost associated with RDTs. Knowledge of RDTs was measured in terms of two variables: general knowledge and clinicians' self-reported level of knowledge. General knowledge was gauged through the statement "Rapid diagnostic tests are fundamental in diagnoses in Kenya today." Responses to this question were coded on a 5-point scale ranging from 1 (strongly disagree) to 5 (strongly agree). Self-determined knowledge was gauged by the question "How would you rate your knowledge of rapid diagnostic tests?" Responses to this question were coded on a 5-point scale ranging from 1 (extensive knowledge) to 5 (no knowledge).

We also measured knowledge with two other variables: potential for replacement of microscopy and types of RDTs clinicians were familiar with. Whether clinicians thought RDTs could replace microscopy was gauged through the statement "RDTs can replace laboratory microscopy tests in areas they are unavailable." Reponses to this statement were coded on a 5-point scale ranging from 1 (strongly disagree) to 5 (strongly agree). Types of technologies clinicians were familiar with was gauged by asking participants about a list of potential diagnostic technologies that are commonly used in diagnostic tests. Knowledge of RDTs was also measured in terms of whether clinicians used RDTs for specific diseases, with open-ended responses.

Besides knowledge of RDTs, we also included variables pertaining to barriers to improve the use of RDTs and the cost associated with RDTs. Barriers to improve RDTs were gauged by the question "Have you encountered any barriers in use of RDTs?" with a yes or no option. The cost

associated with RDTs was gauged with an open-ended question, "What amount of money would be considered as low-cost RDTs by your patients?"

**Development of Survey Instruments**

A short 19-question survey with both open- and closed-ended questions was designed to minimize respondents' time commitment (to avoid fatigue) while covering potentially important factors for RDT design. During the pilot testing of the survey, the items' reliability was assessed using the Cronbach's alpha reliability parameter, which assesses the internal consistency or average correlation of items in a survey. Two constructs were assessed: 1) usability (i.e., ease of use) and acceptability (i.e., probability of use) of RDTs in Kenya, and 2) adoptability (i.e., appropriateness of the RDTs to complement symptom-based or other local diagnostic criteria) of RDTs in Kenyan medical settings. Raw scores were used since all the scales used the same points. The usability and acceptability scale had a raw Cronbach's alpha coefficient of 0.81, which is above the 0.7 cutoff value for acceptance [1]. Deleting the item "Disease known" would have improved the coefficient to 0.93, which implies that the item was somehow unrelated to the construct measured or was too general and therefore skewed the construct. We retained the item because of its importance in eventually informing disease priorities in the design of the RDTs, but interpreted the results with this coefficient in mind. The adoptability scale had a lower reliability coefficient of 0.23, with insignificant improvement when deleting any item. Adoptability is a complementary factor and is compounded by questions of technology trust, which were not part of this study. We can use data from the current study to inform data collection in follow-up studies, including technology trust.

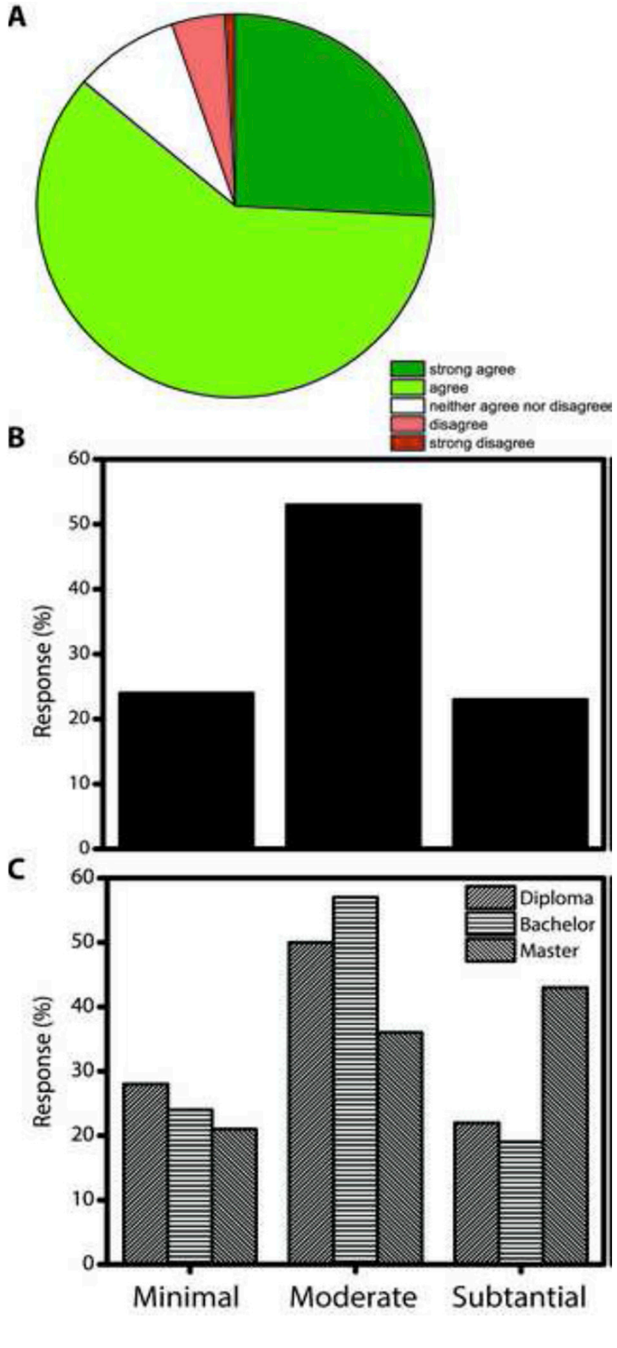


**Figure S1.** Clinician's general RDT knowledge in Kenya. (**A**) Strength of agreement with the statement "RDTs are fundamental in diagnoses in Kenya today." Green: agree and strongly agree; red: disagree and strongly disagree; white: neither agree nor disagree. (**B**) Clinicians' self-reported knowledge level. Substantial = has advanced knowledge, can train others; moderate = can effectively use RDTs; minimal = requires further instructions. (**C**) Knowledge level by clinician education level.

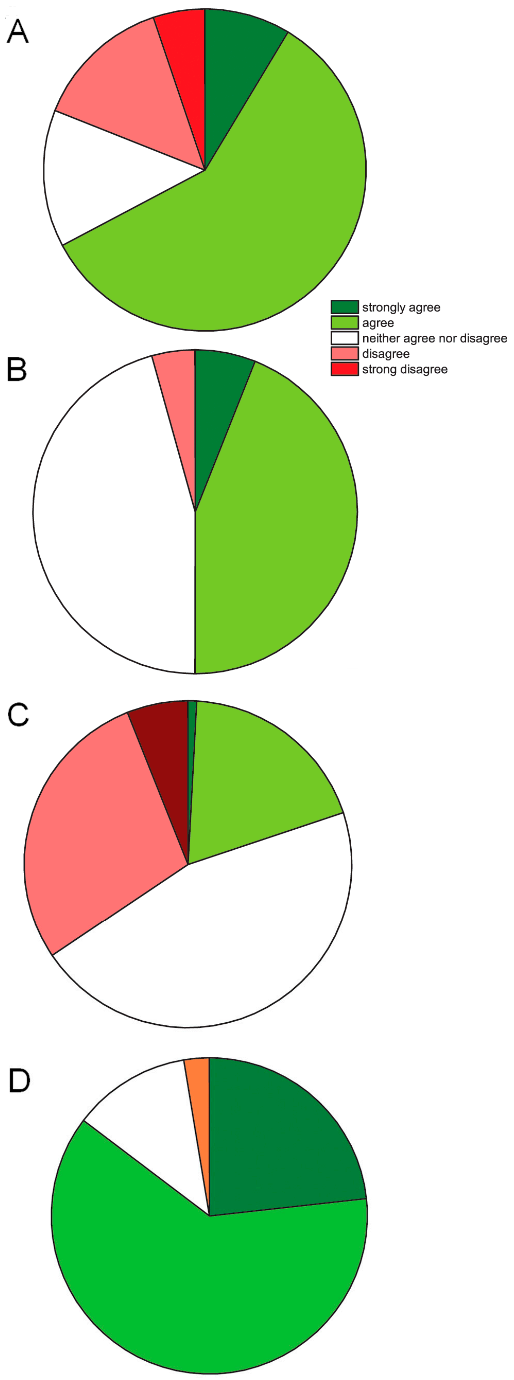


**Figure S2.** Summary of responses to qualitative questions. (A) Strength of agreement with the statement "RDTs can replace microscopy." (B) Strength of agreement with the statement "The results given by RDTs are accurate." (C) Strength of agreement with the statement "I would still prescribe medication for a patient with a negative RDT." (D) Strength of agreement with the statement "RDTs can make health care more affordable in Kenya." Legend: Green: agree and strongly agree; red: disagree and strongly disagree; white: neither agree nor disagree.

## Reference

1. Nunnally, J.C. *Psychometric Theory*, 2nd ed.; McGraw-Hill: New York, NY, USA, 1978.

© 2017 by the authors. Submitted for possible open access publication under the terms and conditions of the Creative Commons Attribution (CC BY) license (http://creativecommons.org/licenses/by/4.0/).
